# Xyloglucan Oligosaccharides Hydrolysis by Exo-Acting Glycoside Hydrolases from Hyperthermophilic Microorganism *Saccharolobus solfataricus*

**DOI:** 10.3390/ijms22073325

**Published:** 2021-03-24

**Authors:** Nicola Curci, Andrea Strazzulli, Roberta Iacono, Federica De Lise, Luisa Maurelli, Mauro Di Fenza, Beatrice Cobucci-Ponzano, Marco Moracci

**Affiliations:** 1 Department of Biology, University of Naples Federico II, Complesso Universitario di Monte S. Angelo, 80126 Naples, Italy; nicola.curci@unina.it (N.C.); andrea.strazzulli@unina.it (A.S.); roberta.iacono@unina.it (R.I.); marco.moracci@unina.it (M.M.); 2 Institute of Biosciences and BioResources—National Research Council of Italy, 80131 Naples, Italy; federica.delise@ibbr.cnr.it (F.D.L.); luisa.maurelli@ibbr.cnr.it (L.M.); mauro.difenza@ibbr.cnr.it (M.D.F.); 3 Task Force on Microbiome Studies, University of Naples Federico II, 80134 Naples, Italy

**Keywords:** glycoside hydrolases, *Saccharolobus solfataricus*, xyloglucan, polysaccharide degradation, archaea

## Abstract

In the field of biocatalysis and the development of a bio-based economy, hemicellulases have attracted great interest for various applications in industrial processes. However, the study of the catalytic activity of the lignocellulose-degrading enzymes needs to be improved to achieve the efficient hydrolysis of plant biomasses. In this framework, hemicellulases from hyperthermophilic archaea show interesting features as biocatalysts and provide many advantages in industrial applications thanks to their stability in the harsh conditions encountered during the pretreatment process. However, the hemicellulases from archaea are less studied compared to their bacterial counterpart, and the activity of most of them has been barely tested on natural substrates. Here, we investigated the hydrolysis of xyloglucan oligosaccharides from two different plants by using, both synergistically and individually, three glycoside hydrolases from *Saccharolobus solfataricus*: a GH1 β-gluco-/β-galactosidase, a α-fucosidase belonging to GH29, and a α-xylosidase from GH31. The results showed that the three enzymes were able to release monosaccharides from xyloglucan oligosaccharides after incubation at 65 °C. The concerted actions of β-gluco-/β-galactosidase and the α-xylosidase on both xyloglucan oligosaccharides have been observed, while the α-fucosidase was capable of releasing all α-linked fucose units from xyloglucan from apple pomace, representing the first GH29 enzyme belonging to subfamily A that is active on xyloglucan.

## 1. Introduction

Hemicelluloses include heterogeneous polymers characterized by a linear backbone with an equatorial configuration highly substituted by short oligosaccharides, monosaccharides, and organic acids. Hemicellulose is the second most abundant polysaccharide in the plant cell wall, representing about 20–35% of lignocellulose biomasses. Its complete degradation requires the simultaneous action of various enzymatic activities due to the heterogeneous composition in monosaccharides, leading to high variability in its structure [1].

Hemicellulose-degrading enzymes belong to the classes of glycosyl hydrolases (GH) and carbohydrate esterase (CE) which are classified in the carbohydrate active enzymes (CAZy, www.cazy.org (accessed on 2 March 2021)) database in functional families based on amino acidic sequences and structure-related similarities [2]. These enzymes have applications in many biotechnological processes, such as biorefineries for the conversion of lignocellulose biomasses in biofuels and bioplastic precursors [3]. Many industrial biomass-degrading processes are performed at high temperatures and extreme pH, to improve the solubility and the availability of organic compounds. In this regard, hemicellulases from (hyper)thermophiles, compared to their mesophilic counterparts, provide many advantages in industrial processes thanks to their high thermal operational stability and tolerance to solvents [4]. In particular, hyperstable carbohydrate active enzymes (cazymes) from (hyper)thermophilic archaea show maximum activity at higher temperature ranges compared to mesophilic cazymes. However, archaeal GHs are poorly represented in CAZy and less studied compared to their bacterial counterparts [5]. Moreover, although most of the GHs annotated in the CAZy database have been characterized by using synthetic substrates, in the framework of biomass saccharification, it is important to test their activity on natural substrates and, more importantly, to investigate their synergistic action on this type of substrate. Cocktails of cazymes need to be improved to achieve a more efficient biotransformation of lignocellulose biomasses [6,7]. Polysaccharides from lignocellulose biomasses can be very heterogeneous in structure and chemical composition, because they can originate from different parts of plants with different degrees of maturation and from a variable number of species.

**Figure 1:**
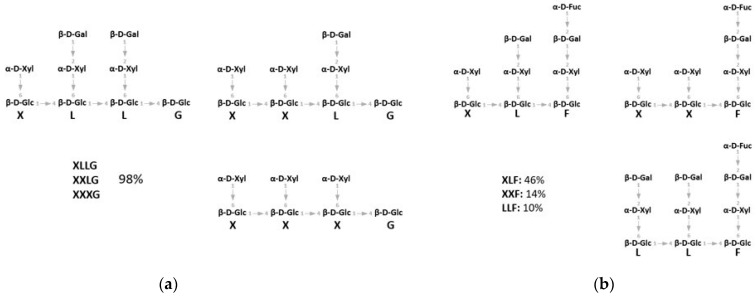
(**a**) Schematic structure of xyloglucan (XG) oligosaccharides (XGOs) from tamarind seeds consisting of a mix of XGOs (XLLG, XXLG, XXXG). (**b**) Schematic structure of XGOs from apple pomace consisting of a mix of XGOs with structure XLF, XXF, LLF (see text for the nomenclature).

Xyloglucan (XG) hemicellulose is present in all land plants. It is among the major polysaccharides of the Type I primary cell wall of all dicots and a great number of monocots [8]. It is composed of a 1,4-β-glucan backbone frequently substituted at O-6 by α-*d*-xylose residue. Xylose is typically the first residue of XG sidechains, which are substituted by up to four glycoside residues, including β-*d*-galactose, which is itself substituted by α-*l*-fucose in some plant species. The sidechains of XG are described by a single letter code, in which G represents the unsubstituted glucose in the backbone, whereas X, L and F represent the glucose residues substituted with α-*d*-xylosyl, β-*d*-galactosyl-(1,2)-α-*d*-xyloside, and α-*l*-fucosyl-(1,2)-β-*d*-galactosyl-(1,2)-α-*d*-xyloside sidechains, respectively (Figure 1) [9,10].

To date, nineteen types of XG sidechain are described, and a large number of enzymes involved in the degradation of XG have been identified and characterized in fungi [8,11] and in Gram-negative and -positive bacteria, such as human intestinal *Bacteroides*, saprophytic *Cellvibrio japonicus* and anaerobic *Ruminoclostridium cellulolyticum* [12,13,14]. In striking contrast, the degradation of XG by archaeal enzymes has been poorly studied. The complete saccharification of XG to component monosaccharides requires the concomitant action of a backbone-cleaving endo-xyloglucanase and exo-glycosidases on the non-reducing ends of XG oligosaccharides (XGOs) [15].

The chemoheterotroph thermoacidophile archaeon *Saccharolobus solfataricus* (previously *Sulfolobus solfataricus*) has also been reclassified for its ability to grow on a variety of carbon sources including sugars, such as polysaccharides (cellulose, starch, dextrin), disaccharides (maltose, sucrose, lactose), hexoses (e.g., d-galactose, d-glucose, d-mannose and l-fucose), and pentoses (e.g., d-xylose and L-arabinose) monosaccharides [16,17,18]. This (hyper)thermophilic microorganism, one of the best-studied in the *Crenarchaeota* phylum, thrives in terrestrial volcanic hot springs and has optimal growth at 80 °C and pH 2–4. In these extreme habitats, lignocellulosic biomass from the nearby vegetation can experience chemical transformation in the hot and acid conditions, providing carbon sources to the microbial community of extremophiles. Consistently, several sequences encoding for GH and mono-, di- and oligo-transporters for sugars uptake are present in the genome of *S. solfataricus* [19,20]. Many of the characterized GHs from *S. solfataricus* showed potential hemicellulolytic activities (GH1, GH2, GH3, GH5, GH12, GH29, GH31, GH36, GH38, GH116), and even some genetic clusters could suggest the ability to hydrolyze hemicelluloses (e.g., *S. solfataricus* P2 Open reading frames (SSO), SSO1353 and SSO1354 encoding for enzymes with β-glucosidase/β-xylosidase and β-glucanase/β-xylanase activity, respectively) [21,22,23,24,25,26,27,28,29,30,31,32]. However, to date, patterns involved in hemicellulose hydrolysis have never been described.

In this work, we tested the activity of three exo-acting GHs from *S. solfataricus* on XGOs from tamarind seeds and apple pomace that we named XGO1 and XGO2, respectively (Figure 1). These enzymes were already biochemically characterized and showed potential activity for the degradation of XGOs: GH1 (LacS) displayed β-glucosidase and β-galactosidase activities, while GH31 (XylS) and GH29 (SsαFuc) showed α-xylosidase and α-fucosidase activity, respectively. LacS and XylS already showed the capability to hydrolyze in a concerted manner short and low-branched XGOs from tamarind seed, while SsαFuc was able to hydrolyze short fucosylated oligosaccharides [21,23,25]. We show here the mechanism of action of the three enzymes on XGOs that allowed us to propose their possible function in vivo.

## 2. Results

### 2.1. Protein Analysis of Recombinant GHs

Recombinant LacS, XylS and SsαFuc were purified with a final yield of 60 mg L^−1^, 1 mg L^−1^ and 1.5 mg L^−1^, respectively. The specific activity of the enzymes was evaluated by using aryl-glycosides as substrates at 65 °C in sodium acetate pH 5.5. LacS had a specific activity of 102 U mg^−1^ and 29 U mg^−1^ on 5 mM p-nitrophenyl-βGlc (pNP-βGlc) and 5 mM o-nitrophenyl-βGal (oNP-βGal), respectively (in the following text we will refer to the LacS units on the pNP-βGlc). XylS had a specific activity of 2 U mg^−1^ on 32 mM pNP-αXyloside, and SsαFuc had a specific activity of 22 U mg^−1^ on 2 mM pNP-αFucoside.

### 2.2. Hydrolysis of XGO from Tamarind Seeds

**Figure 2:**
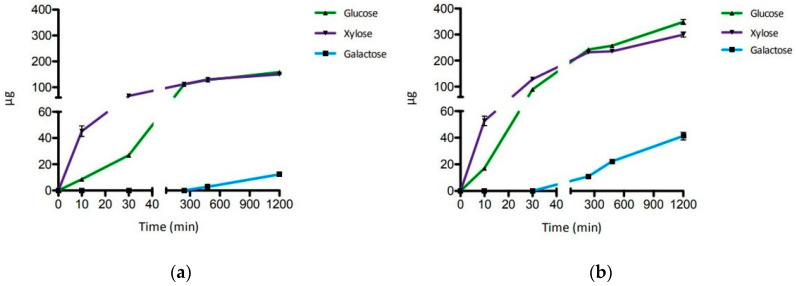
Time course of the enzymatic hydrolysis of XGO1. (**a**) LacS 2.2 U and XylS 0.1 U. (**b**) LacS 18 U and XylS 0.1 U. Error bars reported represent standard deviations.

For testing, we have used XGOs from tamarind seeds (XGO1) that were composed of XXLG, XXXG, and XLLG according to XG nomenclature (Figure 1). The simultaneous and singular activity of LacS, at two concentrations, and XylS on XGO1 have been investigated after 20 h of incubation at 65 °C in sodium acetate buffer, pH 5.5 (Table 1). Moreover, the synergistic activity of the two enzymes was also evaluated after incubations of 10 and 30 min and 4, 8 and 20 h, in the same conditions (Figure 2). Firstly, the activity on XGO1 was evaluated by using 2.2 U for LacS and 0.1 U for XylS. Their simultaneous activity showed the release of galactose, glucose and xylose (12.3 µg, 158.3 µg and 149.7 µg, respectively) (Table 1; Figure S1). XylS alone catalyzed the release of 89.1 µg of xylose, while, in combination with LacS, the amount of xylose increased up to 149.7 µg. LacS action did not lead to any product when acting alone on XGO1, and only the use in synergy with XylS allowed the release of the glucose and galactose. Xylose and glucose were detectable after 10 min, while galactose became detectable only after 8 h of incubation (Figure 2a, Figure S2 and Table S1). Thereafter, in order to understand if increased amounts of LacS could improve the efficiency of hydrolysis of XGO1, we used about eightfold more LacS (18 U), keeping constant the XylS units. Even in this case, LacS alone did not produce detectable amounts of galactose and glucose. However, the amounts of all the monosaccharides released (Table 1, Figure S3) were significantly increased, by 3.4, 2.2, and 2 times, for galactose, glucose and xylose, respectively. The time course of the synergistic action of the two GHs (Figure 2b, Figure S4 and Table S2) showed that at all the incubation times, the amounts of monosaccharides detected were higher if compared to those obtained with 2.2 units of LacS (Figure 2a), and that galactose could be detected after 4 h of incubation. Moreover, release of the monosaccharides increased over time until 20 h. Although this process was not complete compared to the acid hydrolysis, probably due to product inhibition of one or both enzymes, it led to a conversion of 10%, 41% and 37% for galactose, glucose and xylose, respectively (Table 1). This is a remarkable bioconversion efficiency, and demonstrates the good processivity and the extreme thermal operation stability of LacS and XylS on this substrate.

### 2.3. Hydrolysis of XGO from Apple Pomace

The XGOs purified from apple pomace (XGO2) were composed of XLF, XXF, and LLF according to the XG nomenclature (Figure 1b) and showed a 70% degree of purity; the major contaminants were represented by arabinan and chitin as reported in the manufacturing notes. The enzymatic degradation of XGO2 was analyzed after 20 h of incubation at 65 °C in sodium acetate buffer (pH 5.5) by combining 0.3 U, 0.1 U and 2.2 U of SsαFuc, XylS and LacS, respectively, together, in pairs, and singly. Table 2 reports the quantitative analysis of the reaction products of the enzymatic and chemical conversion. The quantifications of the single monosaccharides by amperometric detector (HPAEC-PAD) showed that the three enzymes could release fucose, galactose, glucose, and xylose from this substrate (Figure S5). When the three enzymes acted simultaneously, xylose and fucose were the predominant monosaccharides released, while galactose and glucose were produced in lower quantities. When SsαFuc acted alone or in combination with the other enzymes the amount of fucose released did not vary significantly, suggesting that the enzyme was able to remove the fucosidic residues from XGO2 with no need of previous or concomitant action by the other two enzymes. Interestingly, the amount of fucose released enzymatically was 90% of that obtained by complete chemical hydrolysis of XGO2, indicating that SsαFuc hydrolyzed the substrate with high efficiency. Instead, xylose, glucose, and galactose were released by the three enzymes as 37%, 10% and 3%, respectively, of the monosaccharides identified after chemical treatment. The simultaneous action of XylS and LacS resulted in higher amounts of glucose and xylose compared to those obtained when the two enzymes acted singly or when each of them were separately combined with SsαFuc, and similar to when the three enzymes acted together.

**Figure 3:**
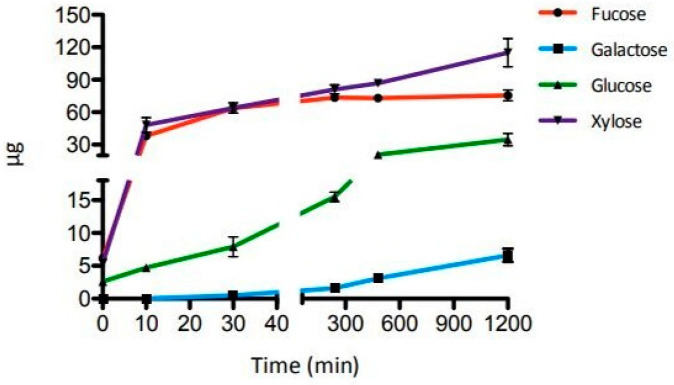
Time course of the enzymatic hydrolysis of XGO2. Error bars reported represent standard deviations.

The time course of the synergistic action of the three GHs was evaluated after incubations of 10 and 30 min and 4, 8 and 20 h. As shown in Figure 3, the amount of galactose, glucose, and xylose released by enzymatic hydrolysis increased until the 20 h mark, while the fucose release reached a plateau after 4 h (Table S3 and Figure S6). A small amount of galactose (0.5 µg) was detectable only after 30 min of incubation. These results confirmed the high efficiency of SsαFuc and the thermal operational stability of the three enzymes at 65 °C on the substrate tested.

## 3. Discussion

In this study, we monitored the release of monosaccharides from XGOs, using different combinations of three GHs from *S. solfataricus* in order to investigate the mode of action of these enzymes on xyloglucan oligosaccharides. The substrates used differed in composition and degree of purity. XGO1, from tamarind seeds which lack α-fucosyl residues, was used to better understand the mechanism of action of XylS and LacS on the X and L components. Instead, XGO2 purified from apple pomace, in which 70% of sidechains showed α-linked fucose residues, was used to investigate the action of SsαFuc on F components and the simultaneous hydrolysis of the α-fucosidase with LacS and XylS.

**Figure 4:**
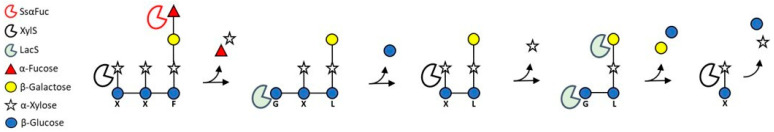
Proposed model of the reaction mechanism on XGOs by XylS, SsαFuc and LacS.

The analysis of the enzymatic hydrolysis of XGO1 confirmed the concerted action of the LacS and XylS exo-glycosidases, catalyzing the processive hydrolysis of α1,6-linked xylose, β1,4-linked glucose and β1,2-linked galactose from the non-reducing end of XGOs. In particular, XylS removed only the first xyloside at the non-reducing end of XGOs, as previously shown on other xylosylated oligosaccharides [21,33] and as reported for other GH31 α-xylosidases on xyloglucan [34]. Therefore, XylS requires the removal of glucose and galactose residues at the non-reducing end to access to the following xyloside residues (Figure 1). LacS, like other GH1 enzymes [34], has broad substrate specificity, promoting the hydrolysis of 1,4- and 1,2-β-*d*-glycosides [23,35,36]. Moreover, an in silico docking study by Kumar and co-workers suggested that a LacS active site could well accommodate short-branched XGOs (GLG) with β-galactose at non-reducing termini in the subsite -1, and the interaction with XGOs could be stronger and selective compared to that of the XG-active β-galactosidase of *C. japonicus* [35]. Indeed, the simultaneous activity of XylS and LacS on both XGO1 and XGO2 produced more xylose compared to the activity of the α-xylosidase alone. In principal, LacS could act on both the β-*d*-linked galactose and glucose residues of XGOs substrates. The substrates used in this work showed galactose at the non-reducing end of XGO branches. Glucose, on the other hand, being decorated by xylose residues, was not accessible to the enzyme (Figure 1). Thus, LacS could operate on galactose residues, while access to bound glucosides would occur only after the previous hydrolysis of 1,6-α-*d*-xylosides promoted by XylS. As expected, LacS alone on XGO1 could not catalyze the release of glucose. However, galactose was not observed either, even by using increasing LacS enzymatic units. This suggests that in order to access to the β1,2-linked galactose, LacS needed partial hydrolysis of the substrate operated by the synergistic and processive action of XylS and LacS itself (Figure 4).

The time courses in Figure 2 show that galactose is released only after 4 h and 8 h of enzymatic incubation, by using 2.2 or 18 units of LacS, respectively. In particular, by increasing the enzymatic units of LacS, even keeping the enzymatic units of XylS constant, the released amounts of glucose and galactose, as well as that of xylose, increased significantly, with an earlier release of galactose resulting in more efficient hydrolysis of XGO1. Higher amounts of LacS offered a greater availability of hydrolyzable substrate to XylS, confirming also, in this case, the synergistic action of the two enzymes.

The GH29 α-fucosidase from *S. solfataricus* released 96% of all α1,2-linked fucose available in XGO2 according to the complete chemical hydrolysis used as control. This indicates that the enzyme was able to access and remove with high efficiency the fucosides decorating XGO2 with no need of prior substrate debranching, indicating the ability of SsαFuc to recognize complex fucosylated oligosaccharides. Most of the α-fucosidases are currently classified in the CAZy database in two major families: GH29 and GH95 [2]. The characterized enzymes of family 95, which also include α-galactosidases, have no activity on *p*NPα-Fuc but are active on α-1,2-linked fucose of XG [37,38]. Instead, according to the classification of Sakurama et al. (2012), the α-fucosidases of the GH29 family were grouped in two subfamilies: GH29A, which are active on *p*NPα-Fuc and several fucosylated oligosaccharides, and GH29B, which are specific for α-1,3- and α-1,4-linked fucosides, but not *p*NPα-Fuc [39]. The GH29B enzyme from *Fusarium graminearum* is active on fucosylated XGOs [40].

SsαFuc belongs to the subfamily GH29A, in which the enzymes display activity toward any α-linked fucose (α-1,2; α-1,3; α-1,4; α-1,6) [41,42]. However, activity on fucosylated XGOs has not been so far observed for GH29A enzymes. We have previously shown that SsαFuc hydrolyzed a short synthetic fucosylated aryl disaccharide α-*l*-Fucosyl(1-3)-α-*l*-Fucosyl-O-*p*NP [25]. Here, we demonstrated for the first time the specificity of SsαFuc for α1,2-linked fucosides on a natural substrate. SsαFuc is the only known characterized GH29A active on fucosylated XGOs. The hydrolytic activity of SsαFuc on XGO1 produced XGOs free of α-fucosyl residues that are a suitable substrate for LacS and XylS.

The data obtained allow us to propose a possible function in vivo of the three GHs from *S. solfataricus*. It is still unclear whether this archaeon could hydrolyze and use XG as a carbon source, as neither xyloglucanase nor XGOs transporters have ever been identified in *S. solfataricus*. To date, the characterized α-xylosidases of the GH31 family are always implicated in XG metabolism and, in the well-characterized XG utilization pathway of *Bacteroidetes* and *C. japonicus*, α-fucosidase activity is involved in XG degradation and utilization [12,14,15,34,37,43,44,45,46,47]. Moreover, XylS and LacS genes mapped in the same locus, in a 50 kb genome region also including the SsαFuc gene [21]. In light of the strict synergism between LacS and XylS, and the high efficiency of SsαFuc to release Fuc from XGOs, the results obtained suggest that the three enzymes could play a role in vivo in the degradation of xyloglucan as an energy source in *S. solfataricus*. Furthermore, from a biotechnological point of view, this study sheds light on the mode of action of the three enzymes on XGOs, laying the foundations for the possible development of a thermostable enzymatic cocktail to hydrolyze XG.

## 4. Materials and Methods

### 4.1. Substrates

Xyloglucan oligosaccharide from tamarind seeds (XGO1) with 98% of purity was purchased from Megazyme (Bray, Ireland). Xyloglucan oligosaccharide from apple pomace (XGO2) with 70% of purity was purchased from Elicityl OligoTech (Crolles, France). The major contaminants are represented by arabinan and chitin.

### 4.2. Enzyme Expression and Purification

The genes encoding for XylS, LacS and SsαFuc were expressed and the proteins purified as previously reported [21,23,25] with the following modifications. The gene encoding for XylS was carried by the plasmid pT7SCII-XylS under the control of an isopropyl -β-D-1-thio galactopyranoside (IPTG)-inducible T7 RNA polymerase promoter and was expressed in *E. coli* BL21 (DE3). The transformed cells were grown in 2 L of Super Broth (SB) supplemented with ampicillin 50 µg mL^−1^. The gene expression was induced with 1 mM of IPTG (Merck, Darmstadt, Germany) at 0.5 OD_600_. XylS was purified by three subsequent cell extract heating steps of 30 min at 55 °C, 65 °C, and 75 °C, followed by anion exchange chromatography. The gene encoding for LacS was carried by the plasmid pET29-LacS under the control of an IPTG-inducible T7 RNA polymerase promoter and was expressed in *E. coli* BL21(DE3). The transformed cells were grown in 2 L of SB supplemented with kanamycin 50 µg mL^−1^ (Merck, Darmstadt, Germany). The gene expression was induced with 1 mM of IPTG at 0.5 OD_600_. LacS was purified by three subsequent cell extract heating steps at 55 °C, 65 °C, 75 °C for 30 min and 85 °C for 10 min, followed by hydrophobic exchange chromatography. The gene encoding for SsαFuc was carried by pGex-SsαFuc plasmid under the control of an IPTG-inducible TAC promoter, and was expressed in *E. coli* RB791 as a fusion protein with Glutathione-S-transferases-tag (GST-tag). The transformed cells were grown in 2 L of SB supplemented with ampicillin 50 µg mL^−1^ (Merck, Darmstadt, Germany). The gene expression was induced with 1 mM of IPTG at 0.5 OD_600_. The recombinant enzyme was purified by affinity chromatography on Glutathione Sepharose 4B resin (GE Healthcare, Chicago, IL, USA) and the removal of GST-tag was obtained by thrombin cleavage.

### 4.3. Protein Analysis of the Recombinant Enzymes

The protein concentration of purified enzymes was measured by the Bradford method using bovine serum albumin (BSA) as standard [48]. Specific activities of the enzymes were measured by using aryl substrates *p*NPα-Xyl, *p*NPβ-Glc, *o*NP-βGal and *p*NPα-Fuc (Carbosynth, St. Gallen, Switzerland) for XylS, LacS and SsαFuc, respectively, in sodium acetate pH 5.5, at 65 °C. The releasing of *p*NP or *o*NP was monitored continuously at 405 nm using a double beam spectrophotometer with a thermal control unit (Varian Cary 100, Agilent Technologies, Santa Clara, CA, USA). The background hydrolysis of the substrates was subtracted by using reference samples identical to the reaction mixtures without enzymes. One unit (U) of activity was defined as the amount of enzyme that released 1 μmol of *p*NP or *o*NP (molar extinction coefficient of 2.08 and 1.2 mM^−1^ cm^−1^, respectively) per minute at the standard conditions.

### 4.4. XGO1 and XGO2 Enzymatic Hydrolysis

A solution of XGO2 (1.1 mg mL^−1^) was incubated with LacS (2.2 U), XylS (0.1 U) and SsαFuc (0.3 U), individually or simultaneously, in 100 mM of sodium acetate buffer, pH 5.5. A solution of XGO1 1.1 mg/mL^−1^ was incubated with LacS 2.2 U or 18 U and XylS 0.1 U, individually or simultaneously, in 100 mM sodium acetate buffer pH 5.5 at 65 °C. “Simultaneous reaction” refers to the assays performed with two or three enzymes used simultaneously. The blank mixtures had identical compositions to the reaction mixtures, using the enzyme storage buffers instead of the enzymes. The assays were performed at 65 °C, and after 10 and 30 min and 4, 8 and 20 h of incubation were stopped through instantaneous freezing in dry ice. All reaction products were analyzed and quantified by high-performance anion-exchange chromatography with pulsed amperometric detector (HPAEC-PAD) analysis (Dionex ICS 3000, Waltham, MA, USA).

### 4.5. Chemical Hydrolysis

XGO1 and XGO2 were dissolved in 2 M of trifluoracetic acid (TFA) at the concentration of 1.1 mg/mL and incubated for 2 h at 100 °C [49]. The reaction solutions were all dried by rotary evaporation to remove TFA. Then, dry pellets were resuspended in distilled water at 1.1 mg mL^−1^ and the drying process was repeated until TFA was completely removed. The monosaccharides composition was determined by HPAEC-PAD.

### 4.6. HPAEC-PAD Analysis

The monosaccharide composition of oligosaccharides XGO1 and XGO2 hydrolyzed via enzymatic and TFA hydrolysis was analyzed by an HPAEC-PAD system equipped with Carbopac PA-100 (Dionex, Sunnyvale, CA, USA). For the assays on XGO1 as substrate, glucose, fucose, galactose and xylose (Merck, Darmstadt, Germany) were used as standards to calibrate the retention time and to build a calibration line at concentrations of 0.5 nmol 25 µL^−1^, 1.0 nmol 25 µL^−1^, and 1.5 nmol 25 µL^−1^, with arabinose (Merck, Darmstadt, Germany) as internal standards at a concentration of 0.5 nmol 25 µL^−1^. For the assay on XGO2, glucose, xylose and galactose were used as standards to calibrate the retention time and to build a calibration line at concentrations of 1 nmol 25 µL^−1^, 2 nmol 25 µL^−1^, and 3 nmol 25 µL^−1^, with fuc as internal standards at concentration of 2 nmol 25 µL^−1^. In total, 25 µL of the samples, supplemented with 0.5 nmol arabinose or 2 nmol of fucose, were injected for the HPAEC-PAD analysis. The elution program consisted of an isocratic elution with NaOH 8 mM for 20 min.

## Figures and Tables

**Table 1:** Enzymatic hydrolysis of XGO1 after 20 h by using LacS and XylS.

Enzymes	Galactose (µg)	Glucose (µg)	Xylose (µg)
TFA hydrolysis ^1^	365 ± 16	835 ± 26	803 ± 9
LacS ^2^ + XylS	12.3 ± 1.1	158.3 ± 1.3	149.7 ± 1
LacS ^3^ + XylS	41.3 ± 2.9	348.9 ± 9.3	299.9 ± 9.6
LacS^1^	ND	ND	ND
LacS^2^	ND	ND	ND
XylS	ND	ND	89.1 ± 9

Trifluoracetic acid (TFA) hydrolysis ^1^: 2 h at 100 °C in 2 M of TFA; LacS ^2^: 2.2 units; LacS ^3^: 18 units.

**Table 2:** Enzymatic hydrolysis of XGO2 after 20 h by using LacS, XylS and SsαFuc.

Enzymes	Fucose (µg)	Galactose (µg)	Glucose (µg)	Xylose (µg)
TFA hydrolysis ^1^	83 ± 2	220 ± 18	346 ± 25	306 ± 11
LacS + XylS + SsαFuc	75.5 ± 5	6.6 ± 1	34.7 ± 5.8	115 ± 13
LacS + XylS	ND	3.15 ± 0.1	35 ± 0.5	118 ± 3.9
XylS +SsαFuc	80.1 ± 14	ND	ND	72.8 ± 9
LacS + SsαFuc	79.5 ± 9	4.7 ± 0.3	24.2 ± 4	ND
SsαFuc	73.3 ± 11	ND	ND	ND
LacS	ND	1.5	13.6	ND
XylS	ND	ND	ND	73.5 ± 11

TFA hydrolysis ^1^: 2 h at 100 °C in 2 M of TFA.

## Supplementary Materials

The following are available online at https://www.mdpi.com/1422-0067/22/7/3325/s1, Figure S1. HPAEC-PAD chromatograms of the enzymatic treatment of XGO1 by using LacS 2.2 U and XylS 0.1 U. Figure S2. HPAEC-PAD chromatograms of the time course of the enzymatic treatment of XGO1 by using LacS 2.2 U and XylS 0.1 U. Figure S3. HPAEC-PAD chromatograms of the enzymatic treatment of XGO1 by using LacS 18 U and XylS 0.1 U. Figure S4. HPAEC-PAD chromatograms of the time course of the enzymatic treatment of XGO1 by using LacS 18 U and XylS 0.1 U. Figure S5. HPAEC-PAD chromatograms of the enzymatic treatment of XGO2 by LacS 2.2 U, XylS 0.1 U and Ss α Fuc 0.3 U. Figure S6. HPAEC-PAD chromatograms of the time course of the enzymatic treatment of XGO2 by LacS 2.2 U, XylS 0.1 U and SsαFuc 0.3 U. Table S1. Monosaccharides composition from the time course of the enzymatic treatment of XGO1 by using LacS 2.2 U and XylS 0.1 U. Table S2. Monosaccharides composition from the time course of the enzymatic treatment of XGO1 by using LacS 18 U and XylS 0.1 U. Table S3. Monosaccharides composition from the time course of enzymatic treatment of XGO2 by LacS 2.2 U, XylS 0.1 U and SsαFuc 0.2 U.
