# Supplementary material for: Xyloglucan Oligosaccharides Hydrolysis by Exo-Acting Glycoside Hydrolases from Hyperthermophilic Microorganism Saccharolobus solfataricus
Source: Int J Mol Sci. 2021 Mar 24;22(7):3325. doi: 10.3390/ijms22073325 (PMC8037949; doi:10.3390/ijms22073325)
Supplement: Supplementary file 1 [file ijms-22-03325-s001.pdf]

Figure S1: HPAEC-PAD chromatograms of the enzymatic treatment of XGO1 by using LacS 2.2 U and XylS 0.1 U

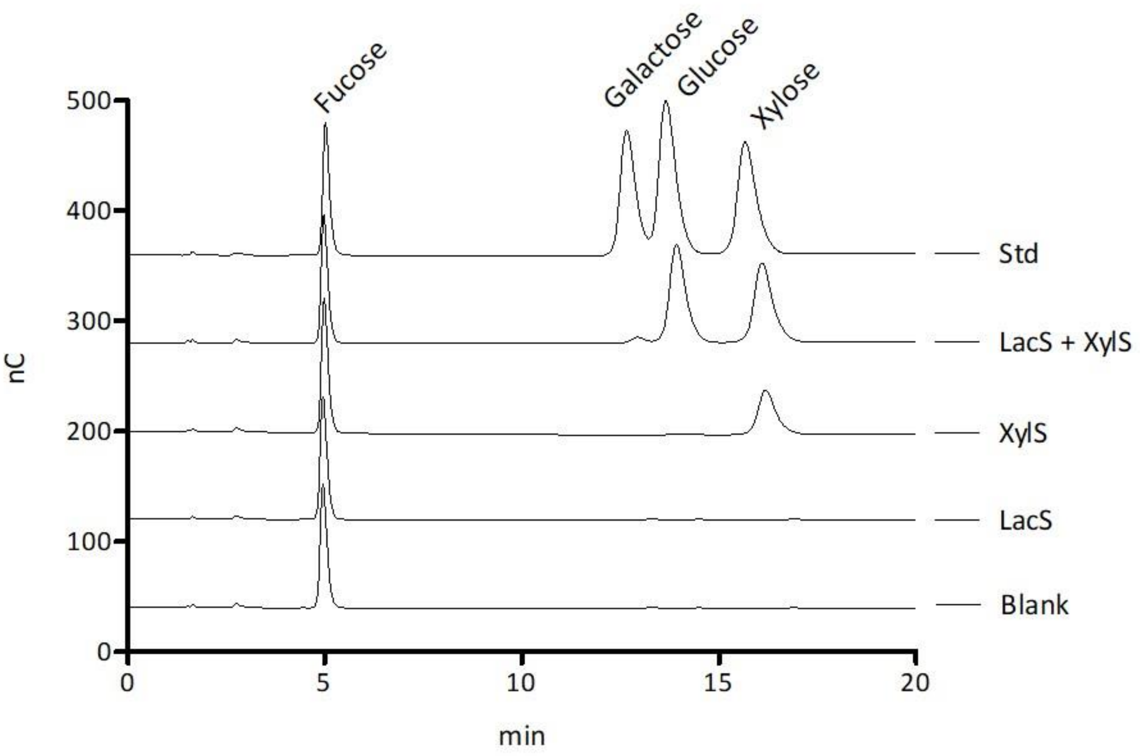

Figure S2: HPAEC-PAD chromatograms of the time course of the enzymatic treatment of XGO1 by using LacS 2.2 U and XylS 0.1 U

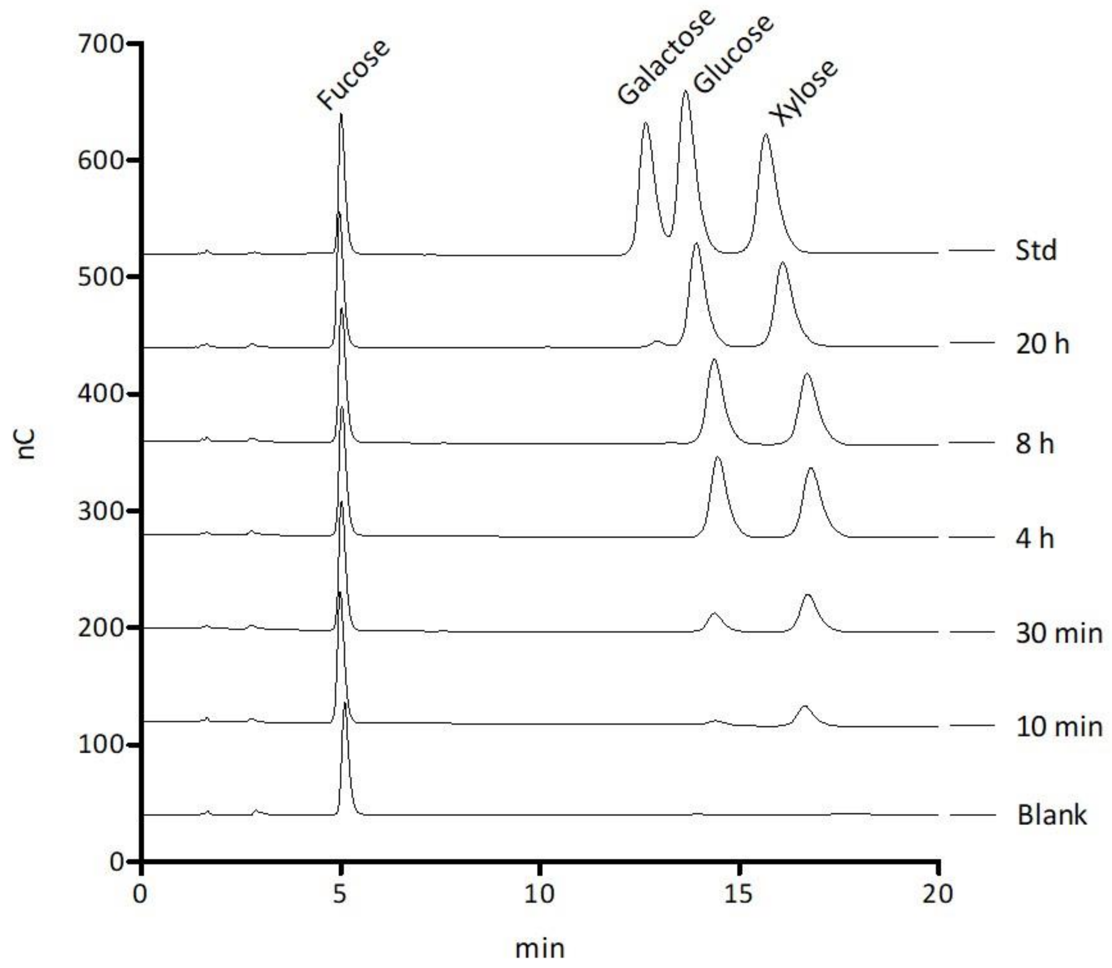

Figure S3: HPAEC-PAD chromatograms of the enzymatic treatment of XGO1 by using LacS 18 U and XylS 0.1 U

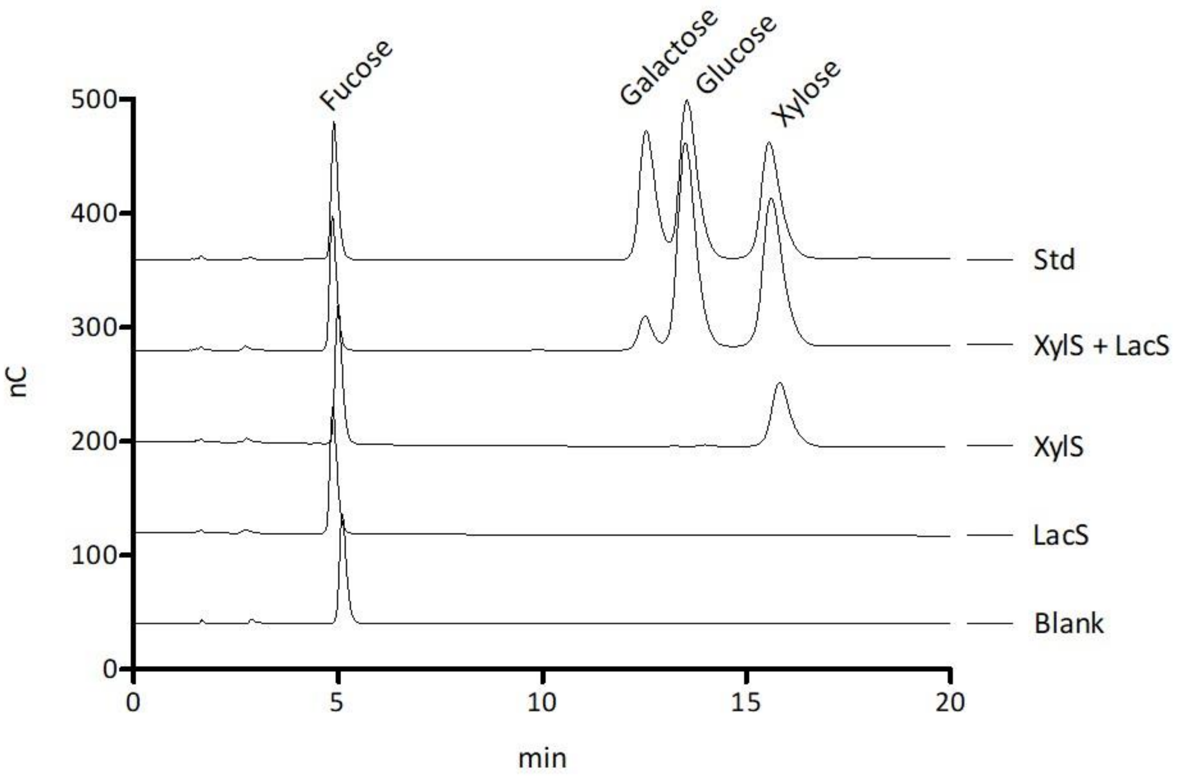

Figure S4: HPAEC-PAD chromatograms of the time course of the enzymatic treatment of XGO1 by using LacS 18 U and XylS 0.1 U

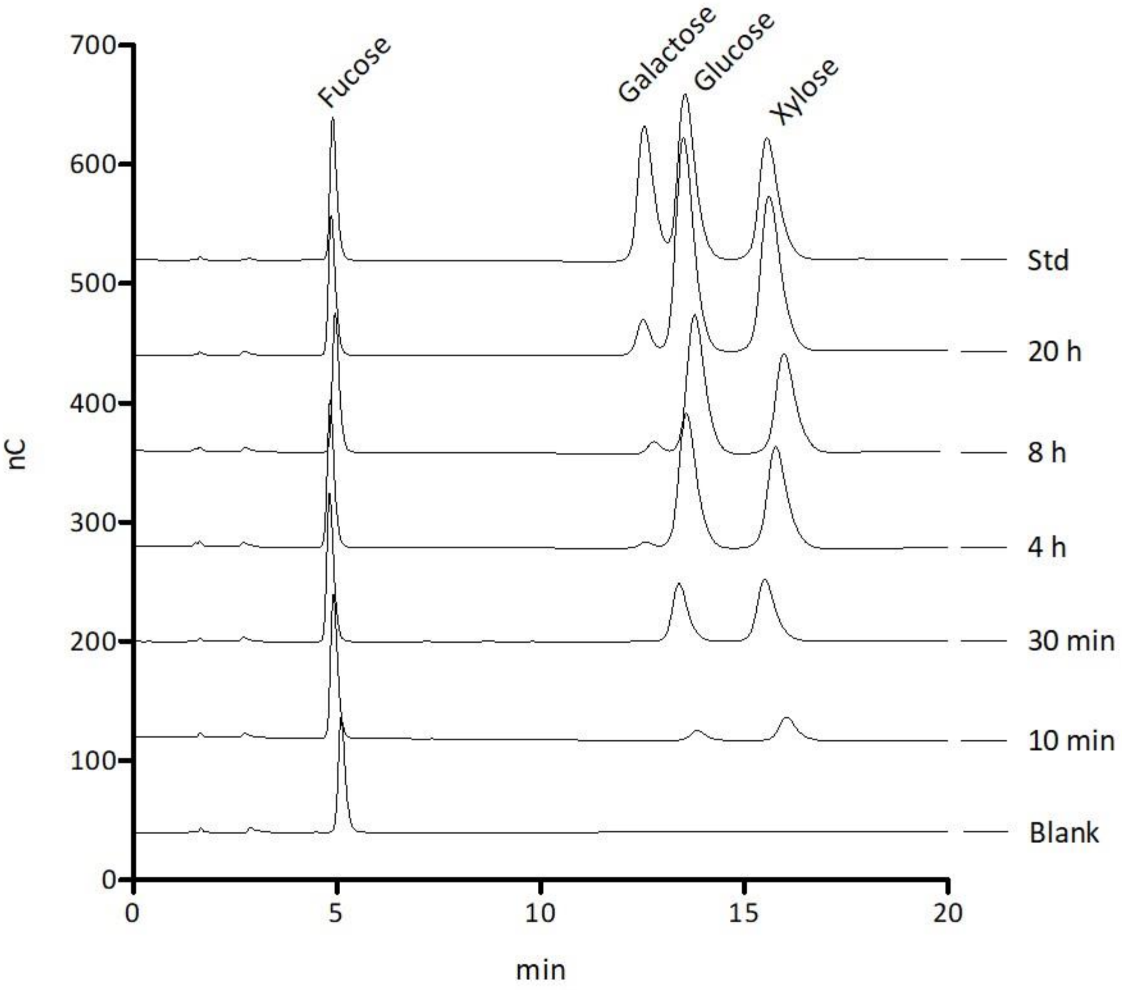

Figure S5: HPAEC-PAD chromatograms of the enzymatic treatment of XGO2 by LacS 2.2 U, XylS 0.1 U and SsαFuc 0.3 U

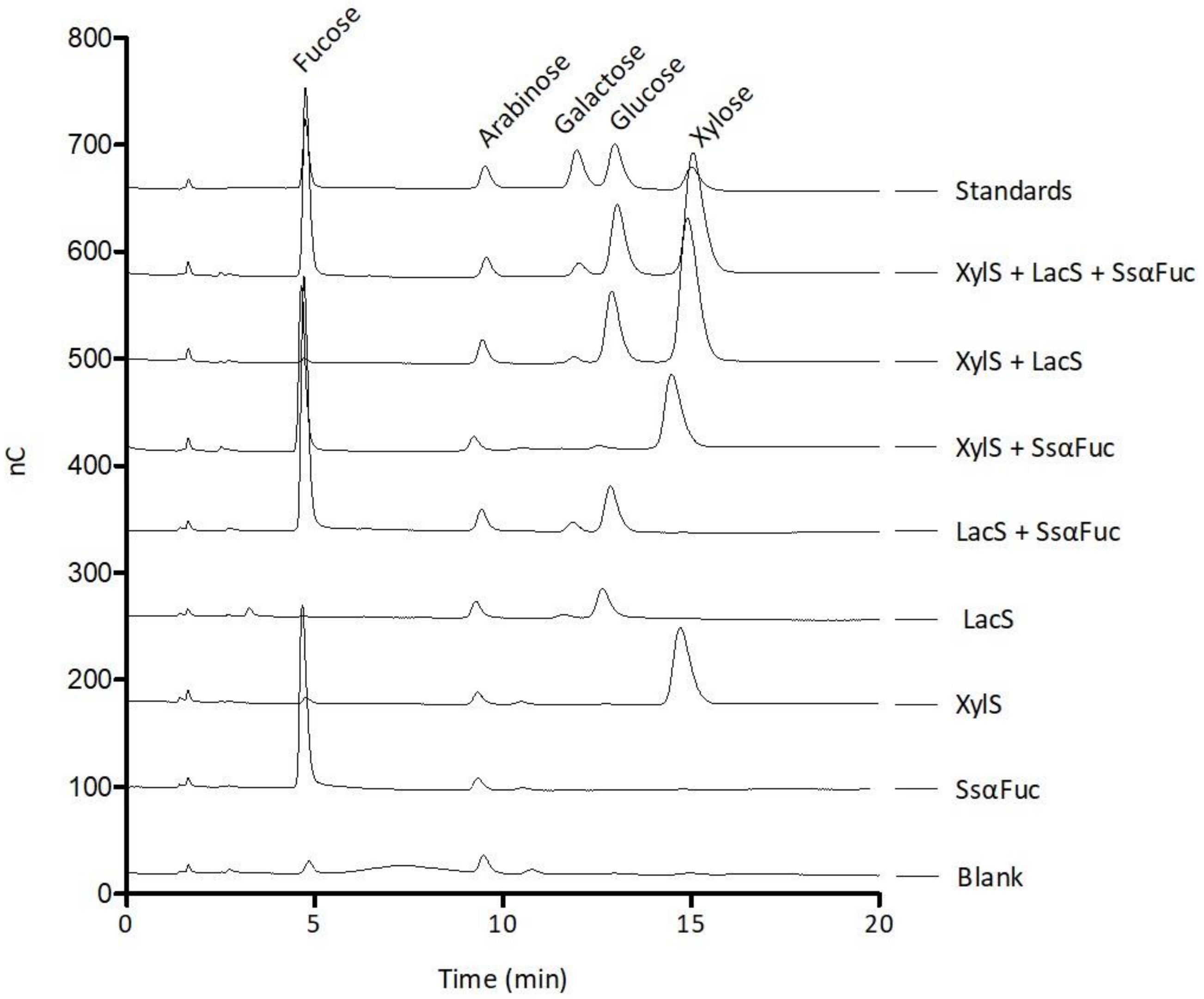

Figure S6: HPAEC-PAD chromatograms of the time course of the enzymatic treatment of XGO2 by LacS 2.2 U, XylS 0.1 U and SsαFuc 0.3 U

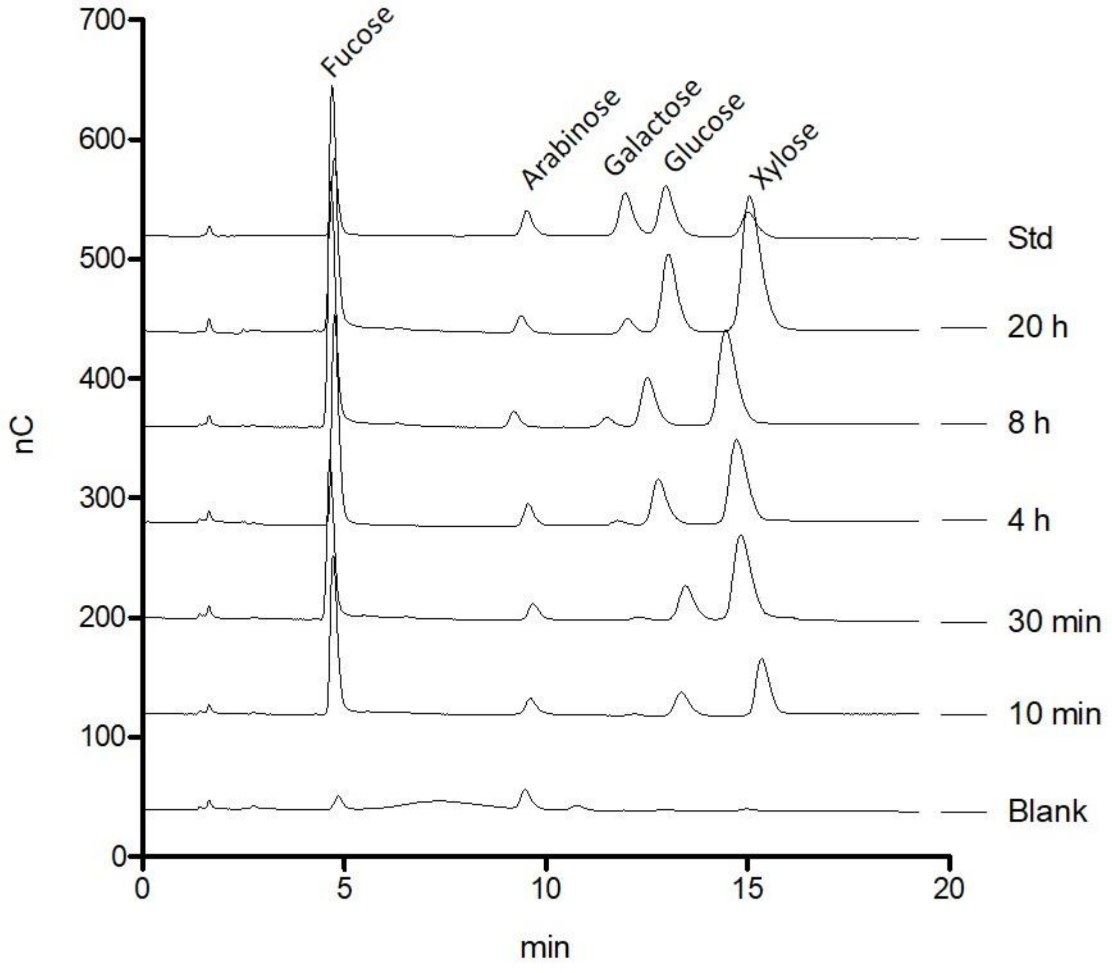

Table S1: Monosaccharides composition from the time course of the enzymatic treatment of XGO1 by using LacS 2.2 U and XylS 0.1 U

Time	Galactose (μg)	Glucose (μg)	Xylose (μg)
T_o	ND	ND	ND
10 min	ND	8.6 ± 1.2	45.2 ± 4
30 min	ND	26.9 ± 0.5	66.7 ± 1
4 h	ND	110.9 ± 2.4	111.7 ± 0.7
8 h	2.9 ± 0.3	128.5 ± 7	130.6 ± 3
20 h	12.3 ± 1.1	158.3 ± 1.3	149.7 ± 1

Table S2: monosaccharides composition from the time course of the enzymatic treatment of XGO1 by using LacS 18 U and XylS 0.1 U

Time	Galactose (μg)	Glucose (μg)	Xylose (μg)
T_o	ND	ND	ND
10 min	ND	17.1 ± 0.3	52.7 ± 3.5
30 min	ND	89.5 ± 3	128.5 ± 3
4 h	10.9 ± 0.6	242.5 ± 6.8	232.1 ± 4
8 h	22.3 ± 0.4	257 ± 0.7	235.6 ± 4
20 h	41.3 ± 2.9	348.9 ± 9.3	299.9 ± 9.6

Table S3: monosaccharides composition from the time course of enzymatic treatment of XGO2 by LacS 2.2 U, XylS 0.1 U and SsαFuc 0.2 U

Time	Fucose (μg)	Galactose (μg)	Glucose (μg)	Xylose (μg)
T_o	6.2 ± 0.4	ND	2.6 ± 0.1	5.4 ± 0.6
10 min	38.2 ± 1.8	ND	4.7 ± 0.4	48.2 ± 6.8
30 min	63.7 ± 2	0.5 ± 0.1	7.9 ± 1.5	64 ± 4.6
4 h	73.5 ± 1.3	1.6 ± 0.2	15.5 ± 0.7	81.1 ± 4.2
8 h	73 ± 1.8	3.1 ± 0.1	20.8 ± 0.1	86.7 ± 3.7
20 h	75.5 ± 5	6.6 ± 1	34.7 ± 5.8	115 ± 13
